# Developing a clinical decision support for opioid use disorders: a NIDA center for the clinical trials network working group report

**DOI:** 10.1186/s13722-020-0180-2

**Published:** 2020-01-16

**Authors:** Gavin B. Bart, Andrew Saxon, David A. Fiellin, Jennifer McNeely, John P. Muench, Christopher W. Shanahan, Kristen Huntley, Robert E. Gore-Langton

**Affiliations:** 1 Division of Addiction Medicine, Hennepin Healthcare, University of Minnesota, 701 Park Avenue, Minneapolis, MN 55415 USA; 2 Department of Psychiatry and Behavioral Sciences, University of Washington, Seattle, USA; 3 Center of Excellence in Substance Abuse Treatment and Education (CESATE), VA Puget Sound Health Care System–Seattle Division, 1660 S. Columbian Way, Seattle, WA 98108-1597 USA; 4 Department of Medicine, Program in Addiction Medicine, Yale University School of Medicine, E.S. Harkness Building A, 367 Cedar Street, Suite 406A, New Haven, CT 06510 USA; 5 Department of Population Health, Section on Tobacco, Alcohol, and Drug Use, NYU School of Medicine, 180 Madison Ave., New York, NY 10016 USA; 6 Department of Family Medicine, Oregon Health & Science University, 3930 SE Division Street, Portland, OR 97202 USA; 7 Section of General Internal Medicine, Boston University School of Medicine, Crosstown Center, 801 Massachusetts Avenue, Boston, MA 02118 USA; 8 Center for the Clinical Trials Network, National Institute on Drug Abuse, National Institutes of Health, Bethesda, MD USA; 9 The Emmes Company, Rockville, MD USA

**Keywords:** Opioid use disorder (OUD), Medications for opioid use disorder (MOUD), Clinical decision support (CDS), Addiction, Drug, Epidemic, Overdose, Prescription

## Abstract

There is an urgent need for strategies to address the US epidemic of prescription opioid, heroin and fentanyl-related overdoses, misuse, addiction, and diversion. Evidence-based treatment such as medications for opioid use disorder (MOUD) are available but lack numbers of providers offering these services to meet the demands. Availability of electronic health record (EHR) systems has greatly increased and led to innovative quality improvement initiatives but this has not yet been optimized to address the opioid epidemic or to treat opioid use disorder (OUD). This report from a clinical decision support (CDS) working group convened by the NIDA Center for the Clinical Trials Network aims to converge electronic technology in the EHR with the urgent need to improve screening, identification, and treatment of OUD in primary care settings through the development of a CDS algorithm that could be implemented as a tool in the EHR. This aim is consistent with federal, state and local government and private sector efforts to improve access and quality of MOUD treatment for OUD, existing clinical quality and HEDIS measures for OUD or drug and alcohol use disorders, and with a recent draft grade B recommendation from the US Preventative Services Task Force (USPSTF) for screening for illicit drug use in adults when appropriate diagnosis, treatment and care services can be offered or referred. Through a face-to-face expert panel meeting and multiple follow-up conference calls, the working group drafted CDS algorithms for clinical care felt to be essential for screening, diagnosis, and management of OUD in primary care. The CDS algorithm was reviewed by addiction specialists and primary care providers and revised based on their input. A clinical decision support tool for OUD screening, assessment, and treatment within primary care systems may help improve healthcare delivery to help address the current epidemic of opioid misuse and overdose that has outpaced the capacity of specialized treatment settings. A semi-structured outline of clinical decision support for OUD was developed to facilitate implementation within the EHR. Further work for adaptation at specific sites and for testing is needed.

## Introduction

In June 2015, the National Institute on Drug Abuse Center for the Clinical Trials Network held a full day workshop on the development of a clinical decision support tool (CDS) for opioid use disorder treatment. The purpose of this meeting was to develop clinical decision support that will lend itself to incorporation into electronic health records (EHRs), and that could assist non-specialist medical providers to identify and manage patients with opioid use/misuse/disorder in general medical settings. Following this full day meeting, a work group was created to sketch out what this CDS would entail. Over a series of conference calls, webinars, and focus groups, recommendations for a CDS were formed. This paper summarizes the final white paper reflecting the recommendations of the expert panel.

Strategies are urgently needed to address the US epidemic of prescription-opioid, heroin and fentanyl-related overdose, misuse, addiction, and diversion. The Mental Health Parity Law [1] and the Affordable Care Act [2] have provided impetus and opportunity to integrate substance use disorder and mental health treatment into general medical settings. Evidence-based treatment options for opioid use disorder (OUD) are available [3], yet there are not enough health care providers and programs that provide medications for OUD (MOUD) in the US to address demand adequately, and there is tremendous need for additional MOUD providers in primary care and other general medical settings.

The Drug Addiction Treatment Act of 2000 allows many prescribers to complete additional training (8–24 h, depending on licensure) and apply for a “waiver” to prescribe buprenorphine for the treatment of OUD. More than 72,000 prescribers are now waivered, yet many of them do not go on to treat OUD [4–6]. Compared to those who do prescribe buprenorphine, waived prescribers who do not are more likely to endorse lack of institutional support as being a barrier. Other identified barriers include lack of staff training, lack of confidence, poor access to clinical guidelines, and time constraints. Similar barriers have been identified as limiting use of extended-release naltrexone (XR-NTX), which does not require special training or a waiver [7, 8]. Tools that can help prescribers overcome these barriers may lead to increased treatment of OUD in general medical settings.

Evolving electronic health record (EHR) data/informatics capacity has led to innovative quality improvement initiatives in health care systems, and these innovations can be leveraged to help address the opioid epidemic and treat OUD. Now is the time to leverage the available technological tools in the EHR to improve screening, identification, and treatment of OUD in primary care settings. The development of a CDS support tool, which will serve precisely this purpose by supporting primary care providers in assessing and managing patients with OUD, is the centerpiece of this document.

## Background

The U.S. Preventive Services Task Force (USPSTF) recommends that clinicians screen adults aged 18 years and older for alcohol misuse and provide persons engaged in risky or hazardous drinking with brief behavioral counseling interventions to reduce alcohol misuse [9]. There is growing support for screening for drug use as well. In September 2019 the USPSTF issued a draft of a new grade B recommendation for screening for illicit drug use in adult primary care patients [10], representing a change from its earlier determination that there was insufficient evidence to support this practice [11]. The 2015 Institute of Medicine (IOM) report “Vital Signs: Core Metrics for Health and Health Care Progress” recommends that a set of measures be incorporated into federally administered health programs that identifies addictive behavior (including tobacco use, drug dependence/illicit use, alcohol dependence/misuse) as a core metric [12].

While more research on the benefits and potential risks of universal screening for illicit drug use is needed, primary care office-based MOUD has a robust evidence base as recently reported by the National Academies of Sciences, Engineering, and Medicine [3]. In the face of rising prevalence of prescription and illicit opioid misuse and skyrocketing opioid overdose deaths, federal, state, local, and private sector efforts are now clearly focused on expanding and improving treatment delivery. In 2015 the Obama Administration released a Presidential Memorandum that calls for improved access to MOUD, training of Federal providers, contractors, and trainees in the identification of patients with OUD, and the provision of treatment or referral for these patients [13]. This memorandum aligns with other efforts to improve the quality of care for patients with OUD. For example, the National Committee for Quality Assurance Healthcare Effectiveness Data and Information Set (HEDIS) measures the percentage of individuals with a diagnosis of alcohol or drug dependence who initiated treatment and who had two or more additional services within the subsequent 30 days [14, 15]. In 2017, the Trump administration declared the opioid crisis a public health emergency and has subsequently issued a nationwide call to action to address the opioid crisis that includes expanded access to MOUD [16, 17].

The Department of Health and Human Services’ National Quality Strategy and Meaningful Use requirements under the EHR Incentive Programs, administered by the Centers for Medicare and Medicaid Services (CMS) and the Office of the National Coordinator for Health Information Technology (ONC), promote continued innovations in electronic health record utilization. In 2016, ONC’s Meaningful Use requirements entered Stage 3, which mandated that EHR information be capable of improving clinical outcomes, care coordination, population health, and patient empowerment. Clinical decision support tools are a recognized means of achieving Stage 3 Meaningful Use goals [18, 19].

## Development process

The NIDA Center for Clinical Trials Network convened a Working Group to develop a CDS that would serve as an evidence-based set of clinical recommendations from experts in the treatment of OUD, for incorporation into EHRs to assist medical providers in identifying and managing patients with unhealthy opioid use in general medical settings. The Working Group held a series of conference calls, webinars, and an in-person meeting from June through November 2015.

The ONC and Centers for Disease Control and Prevention (CDC) are working in parallel on developing clinical decision support for prescribing opioids for pain; that distinct effort is intended to assist providers in making decisions regarding the use of opioids for chronic pain. That work and the work reported herein are likely to complement each other.

The model developed by the NIDA Clinical Decision Support for Opioid Use Disorders Working Group builds upon a NIDA National Drug Abuse Treatment Clinical Trials Network (CTN) study seeking to develop and validate a 4-item screen and a two-stage screening and brief assessment tool to assess primary care patients for tobacco, alcohol, prescription drug, and illicit substance use and problems related to their use (Tobacco, Alcohol, Prescription medications, and other Substance [TAPS] tool) [20]; upon a 2011 Clinical Decision Support for substance use disorder (SUD) Expert Consensus Meeting; and on the resulting CDS for SUD model that was presented at the 8th Annual Conference of the International Network on Brief Interventions for Alcohol and Other Drugs (INEBRIA), September 23, 2011 and the College on Problems of Drug Dependence (CPDD), 2012.

Clinical content in the CDS is based upon the TAPS validation study, research evidence on motivational interviewing, and principals of shared decision making. Several extant resources were also utilized including: *ASAM National Practice Guideline for the Use of Medications in the Treatment of Addiction Involving Opioid Use* (which was published while the Working Group was developing its algorithm), *American Psychiatric Association Practice Guideline for Treatment of Patients with SUD*, SAMHSA TIPs 40 and 43 (TIP 63 was published after the workgroup completed its task), and *VA/DoD Clinical Practice Guideline for Management of SUD* [21–25].

The Working Group provided subject matter expertise and developed clinical content for clinical decision support. The Working Group conducted a workshop on the CDS at the 12th Congress of INEBRIA to obtain audience feedback on the clinical workflow. Further stakeholder webinars for primary care providers and addiction specialists were held in late 2015 and early 2016. The goal of these webinars was to review the CDS algorithm with addiction specialists and primary care providers and to make revisions based on their input. The Working Group acknowledged that future work will require e-specification to capture data for analysis of quality metrics, integration into EHR systems, and feasibility/usability testing.

The Substance Abuse and Mental Health Services Administration (SAMHSA), Health Research Services Administration (HRSA) and the Office of the National Coordinator for Health Information Technology (ONC) were engaged in the process and shared information and expertise to facilitate this project. SAMHSA presented information to the Working Group about SAMHSA’s Clinician Education activities and Health Information Technology activities. HRSA provided information about HRSA Health Center Controlled Networks and Health Information Technology efforts. ONC gave presentations to the Working Group on the National Quality Framework and the Opioid Prescribing Pilot Project.

A narrative description of the clinical guidance is conveyed in this document, and the corresponding schematic is shown in Additional file 1: Figure S1. The schematic covers areas of screening, patient assessment, goal setting, shared decision making for treatment options, MOUD treatment with buprenorphine, naltrexone, or referral for methadone, and care considerations common to all patients with OUD. The clinical decision support creates a semi-structured framework that can be adapted to local EHR environments and staff workflows.

## Clinical decision support for opioid use disorders

The workflow begins with screening and initial assessment but is conceptualized and designed so that there could be multiple entry points once embedded in an EHR. For example, a patient could present stating that she/he wanted to stop misusing prescription opioids, and the clinical provider could begin with the ‘assessment of risk level’ or, ‘assessment for dependence’, rather than initial screening questions. The clinician could have already made a determination to treat the patient with buprenorphine/naloxone and could enter the CDS at the point that specifically supports initiating treatment with this medication. Also, different healthcare systems utilizing a different screening and assessment tool could adapt and use the clinical content of the rest of the workflow with their system’s screening tool. For example, if a health care system was already using another screening tool (e.g., the WHO ASSIST or DAST-10) to identify ‘moderate-risk’ or ‘high-risk’ use, the CDS could be initiated accordingly.

## Screening and initial assessment including readiness to change

The TAPS Tool can be administered by a clinician or self-administered by patients on an electronic device such as a tablet or computer. A score of 1 or greater on the TAPS Tool for heroin or prescription opioids indicates that a patient has problem opioid use and may benefit from intervention by the clinician.

Following a positive screen, the initial step is for the clinician to provide brief motivational counseling. While brief counseling alone may be insufficient to reduce unhealthy drug use [26–28] and certainly if a patient already has moderate or severe OUD, it can be an effective means of raising concern and establishing the patient’s goals regarding treatment. In the primary care setting, we recommend using a brief negotiated interview (BNI) [29] format, which consists of raising the subject, providing feedback on the screening score, recommending cessation or reduction of opioid use, assessing readiness to change, and asking patients if they are ready to set a goal for changing their substance use. These steps can be conducted in as little as 3–5 min and are outlined below, with examples of what the provider might say during each section of the discussion. Providers could seek training in brief intervention/motivational interviewing to be most effective in these conversations and may find it helpful to refer to the BNI manual for guidance [29].

1. Raise the subject
  Ask permission: “Would you mind if we spend a few minutes talking about your use of [name drug(s)]?
  Engage the patient: “I want to talk about how it’s affecting you and how we might be able to help.”
2. Provide feedback
  Review screening data and express concern: “The substance use questionnaire you completed indicates that you are using [name drug] in a way that is harmful to your health. It can interfere with the treatment of your other medical problems [connect to reason for today’s visit] and puts you at high risk for developing a severe addiction or dying from an overdose.”
3. Recommend cessation/reduction
  “Given the health problems that come with [drug] use, my recommendation is that you stop or cut down.”
  “I’m here to help you, and I believe this is an important medical problem. In order to help you with this, I would like to talk with you a bit further about your goals, and then discuss options for how I can best support you.”
4. Assess readiness and confidence. Self-rated readiness and confidence have both been associated with substance use outcomes for alcohol and drug use [30–32]. Both readiness and confidence can be quickly assessed on a 10-point scale using single-item questions, and have been validated in this format [33].
  “On a scale of 1 to 10, where 1 is ‘not at all ready’ and 10 is ‘very ready’, how ready are you to change your drug use?”
  “On a scale of 1 to 10, where 1 is ‘not at all confident’ and 10 is ‘very confident’, how confident are you in your ability to change your drug use?”
5. Enhance motivation by asking the patient why her/his readiness and confidence ratings are not lower.
  If patient says > 2, ask “Why did you choose that number and not a lower one?”
  If patient says 1, ask “What would it take for that one to turn into a two?” Or “What would have to happen for you to feel ready/confident? How important would it be for you to prevent that from happening?”
6. Ask patient to set a goal for changing her/his use:
  Summarize the conversation: “So what I’ve heard from you is that you are [summarize readiness/confidence] to make a change in your [drug] use.”
  Reinforce autonomy, ask patient to set a goal: “While you know that my recommendation is to stop using, it’s really up to you to decide if you want to make a change, and I’m here to work with you on anything you feel ready to do that will improve your health. Do you feel like you can set a goal of making any changes in your use, whether it’s stopping, using less, or using more safely?”

The subsequent steps depend on the patient’s goal regarding her/his opioid use. If the patient’s goal is to *stop* use, the TAPS score can be used to indicate whether use is moderate-risk or high-risk.

- If moderate-risk use: Set a clear goal regarding how she/he will stop/reduce her/his opioid use; provide overdose prevention education and prescribe naloxone; and arrange for a follow-up visit (within 2 months is recommended).
- If high-risk use: Assess for an OUD based on DSM-5 or ICD-10 criteria.

If the patient’s goal is *no change* in opioid use, the clinician should acknowledge the patient’s autonomy and offer assistance whenever the patient is ready to make a change. Educational materials and location-specific drug treatment and harm reduction resources can be provided. The clinician should reassess current opioid use and goals at the next medical visit.

If the patient is *unsure* about her/his goal, the provider can offer additional motivational counseling and make plans to revisit this in a follow-up visit:

- Ask about the pros and cons of changing opioid use. “What are some things that would be better if you were to stop using [drug]?” “What are some things that would be worse, or that you would miss if you were to stop using [drug]?”
- Discuss barriers and facilitators of making a change: “What are some of the things that might support you in changing your [drug] use?” “What are some of the things that might get in your way?”
- Schedule a follow-up visit (within 2 months is recommended) to discuss further.

For all patients with a positive screen for opioid use, some basic clinical care is recommended to address common comorbidities and health risks [34, 35]. These actions are listed in the ‘Clinical Care Module.’ While not all of these items will necessarily be addressed in a single visit, it is desirable to complete them all in a timely fashion (e.g., within 6 months of the initial identification of unhealthy opioid use). Many items may have been completed previously, in which case the information should already exist in the EHR (and can be pre-populated or noted within the CDS). Most of the items are straightforward and are listed in the CDS algorithm—for others, suggested language/instruments are below.

- Screening for tobacco, alcohol, and other drugs is already done if practices are using the TAPS Tool and can be presented as part of the CDS and reviewed by the provider.
- Assessment of IDU: “Have you ever used any drug by injection?” If yes, “When was the last time you injected?”
- Drug treatment history: “Have you ever been in treatment for drug/alcohol use?” If yes, “Are you currently in treatment for substance use?”
- Screening for common health and mental health problems: HIV, viral hepatitis, depression (e.g., PHQ-2/9) [36]; review chart and ask about chronic pain. Screen women of childbearing age for pregnancy and discuss contraception. Discuss that fertility may increase when patients are treated for OUD, as health improves and menstrual cycles normalize [37, 38.]
- Screen women for intimate partner violence using a brief validated instrument (e.g., HITS, HARK, STaT) [39].
- Overdose education and naloxone prescription [40].

## Shared decision making

Following initial screening and assessment of readiness to change, patients who are diagnosed with a moderate or severe OUD can be engaged in a shared decision-making process to discuss treatment options. Shared decision making is a process whereby the provider and patient collaborate to make decisions about the course of care [41]. This differs from an informed decision-making process; rather than simply providing information to the patient about treatment options and leaving the patient to be the sole decider of care, shared decision making allows the knowledge and advice of the provider to be offered to the patient so that she/he may incorporate this information into their own decision-making process. Together the patient and provider reach a consensus about the treatment plan.

Patients with OUD can learn that it is a highly treatable chronic disorder that will require ongoing management. Medically supervised withdrawal is not sufficient for the treatment of OUD, although it may serve as a bridge to initiating ongoing care. The essential treatment question for shared decision making in OUD is one of MOUD or behavioral treatment approaches. For an MOUD approach, which medication option is the best fit for the patient? While the data strongly indicate superiority of MOUD approaches versus behavioral approaches alone for outcomes such as relapse to drug use and mortality, providers ideally will discuss all options with patients [42]. Information that may be required to facilitate shared decision making includes discussing the patient’s goals, effects of various treatment approaches on mortality, HIV and viral hepatitis acquisition/transmission, quality of life, comparison of the risks and benefits of available medications (including cost, frequency of clinic visits, and side-effects), and the impact of treatment on comorbid illnesses.

Resources to be drawn upon to facilitate shared decision making for OUD likely exist within health systems or are readily available in multiple formats (e.g., brochures, web sites, interactive educational, etc.) and include: nature of addiction as a chronic disease, the risks of drug use in terms of overdose, HIV, viral hepatitis and other medical consequences, description of medications (e.g., SAMHSA Facts patient brochures). Materials which may need development by institutions (or by the CDS workgroup) or referenced from the ASAM National Practice Guideline for the Use of Medications in the Treatment of Addiction Involving Opioid Use include medication comparison charts which allow patients to do a side-by-side comparison of factors that may be of importance from the patient perspective. Table 1 is a basic example of the types of comparative information patients may want to know as they make a decision:

**Table 1:** Comparative information for patients to make a decision

	Methadone	Buprenorphine	Oral naltrexone	Extended release naltrexone
Route	Oral daily	Sublingual daily or every other day	Oral daily or three times per week	Intramuscular injections every 28 days
Constipation	++	++	−	−
Sexual dysfunction	++	+	−	−
Physical dependence	++	++	−	−
Sweating	++ (rare)	−	−	−
Starting/Stopping	No lead-in abstinence Gradual taper	Either lead-in abstinence or no lead-in abstinence if in opioid withdrawal at time of induction Gradual taper	Lead-in abstinence (2–10 days) No taper	Lead-in abstinence (2–10 days) No taper
Weight gain	−	−	−	−
Sedation	±	±	−	−
Bone/joint pain	−	−	−	−
Dental problems	−	−	−	−
Opiate effect	++	+	−	−
Interferes with pain management using opiates	−	±	+	+
Drug interactions	HIV medications Phenytoin Rifampin Carbamazepine Benzodiazepines Alcohol	Atazanavir Benzodiazepines Alcohol	Opioid analgesics	Opioid analgesics-
Risk of overdose	+	± (with benzodiazepines)	−	−

Patients should be informed that there is no clearly defined length of MOUD. The literature indicates that the longer people take medication the less likely it is that they will return to regular opiate use [43]. Patients should be informed of the high relapse rate when medications are stopped (even with gradual tapering) and that there are few predictive factors that can help a practitioner identify patients who are likely to succeed following medication discontinuation. Furthermore, patients should be informed of the risk of overdose and death following MOUD discontinuation due to loss of tolerance.

Patients should know that specialized treatment facilities are available to provide medications and that these settings may have additional services not available in a general medical setting (e.g., group therapy, vocational programs) but that these programs may also require more intensive attendance. Discussion of the privacy of treatment records between general medical settings and specialty settings or specialty provider in general medical settings (i.e., 42 CFR Part 2) should also occur.

Patients may need time to weigh their treatment options. For patients diagnosed with OUD, it is recommended that a follow-up appointment to discuss treatment choices be scheduled within approximately 2 weeks after diagnosis.

For the patient and/or provider who determine that referral for treatment is the best option, the CDS will prompt for follow-up to make sure that a referral appointment was made and completed. For patients and providers choosing intervention within the general medical setting, available medications are described below.

## Office-based buprenorphine

Food and Drug Administration approved buprenorphine formulations for the treatment of OUD can be considered for those with moderate to severe OUD with current physiologic dependence. They may also be considered for those without physiologic dependence who are high risk for relapse [24].

### Evaluation phase

In anticipation of initiating buprenorphine treatment, a number of clinical issues should be considered as outlined below:

#### Is the patient pregnant?

If the patient is pregnant or planning to become pregnant, the clinician should discuss the evidence supporting the use of buprenorphine and methadone during pregnancy and breastfeeding. This should include the availability of resources for pregnant women in the clinician’s office compared to those available in OTPs [44].

#### Does the patient have chronic pain that requires opioid agonists?

If the patient has chronic pain, the clinician should discuss the moderate analgesic properties of buprenorphine with the patient. For patients with mild to moderate chronic pain, clinicians can attempt a trial (2–4 weeks) of buprenorphine treatment to determine if adequate analgesia is obtained. If the patient and clinician opt not to attempt to such a trial or if such a trial is unsuccessful, the patient should be referred to an opioid treatment program (OTP) for consideration of methadone which may provide additional analgesia and can allow for the use of full opioid agonists for pain if deemed necessary and appropriate [23].

If the patient is interested in initiating treatment with buprenorphine, the clinician should assure adequate coverage for medication, visits, counseling, urine drug screening, blood testing including baseline liver tests, the ability to safely store medication, and check for any contraindication to the use of buprenorphine.

### Induction phase

Once the decision has been made to initiate treatment with a buprenorphine formulation, buprenorphine induction can occur as outlined in the Substance Abuse and Mental Health Administration’s Treatment Improvement Protocol #40 [24] with certain modifications as outlined in the ASAM National Practice Guideline for the Use of Medications in the Treatment of Addiction Involving Opioid Use [21]. These include the use of co-formulated buprenorphine/naloxone (bup/nx) products, consideration of unobserved (e.g., home) induction when both the clinician and patient are experienced in the use of buprenorphine, and a target of 16 mg (or equivalent doses for some proprietary products with differing bioavailability) on the second day of treatment.

### Stabilization phase

Patients are seen for office visits at least weekly in the early phase of treatment to allow for clinical assessment and dose adjustment [23, 24].

### Treatment phase

During the treatment phase, patient visits can be extended to twice monthly or monthly depending on evidence of clinical stability. During visits, clinicians should provide medication management, assess patient response to treatment, obtain urine drug screens as clinically indicated, and adjust clinical services based on that assessment. If it is determined that the patient is adherent to the prescribed treatment plan, is abstinent or using illicit opioids at a level that does not interfere with function, then it is appropriate to continue treatment with modifications to achieve a long-term goal of abstinence from illicit drug use.

If the patient is adherent to the prescribed treatment plan, but reports intermittent or ongoing low-level use of illicit opioids at a level that interferes with function, the clinician should assess potential problems in the following domains: medication adherence, buprenorphine dose to assure cross tolerance and narcotic blockade, unaddressed triggers to ongoing drug use such as negative influences from people, places and things, comorbid substance use (e.g., alcohol, non-medical use of benzodiazepines), and unmet psychosocial counseling needs.

If the patient is not adherent to the prescribed treatment plan, has not been able to achieve abstinence and is persistently using illicit opioids at a level that interferes with function after the clinician and the patient have made repeated attempts to address the issues outlined including adjustments in medication dose and counseling, then consideration should be made regarding a transfer to an office-based practice or outpatient treatment program that is able to provide greater structure and resources.

In determining the appropriateness of a patient for a specific office-based practice, the clinician should consider her/his expertise and available resources to address the following comorbid conditions: comorbid substance use and or substance use disorders, comorbid pain, and untreated psychiatric comorbidity. In addition, some patients may require closer monitoring and more frequent visits to monitor urine toxicology results, ensure medication adherence, avoid diversion, and provide adequate counseling [24].

## Naltrexone

Naltrexone is a reasonable option for patients who have an opioid use disorder but do not have current physiologic dependence. Such patients could have recently completed medically supervised withdrawal from opioids. They could have recently been incarcerated and gone through withdrawal in a penal setting. They could have stopped opioids temporarily on their own.

If the patient is willing to take naltrexone, there are some next steps before starting the medication. First assure that baseline liver tests have been obtained since oral naltrexone has an FDA boxed warning for liver injury. It is generally suggested by experts that, if the patient has baseline transaminases > 5X upper limit of normal, naltrexone should be used with extreme caution and careful monitoring. Second, obtain a urine drug screen for opioids, oxycodone, methadone, and buprenorphine. Because naltrexone, as a competitive antagonist, can cause precipitated opioid withdrawal in patients not fully withdrawn or who have remaining opioids on their mu-opioid receptors [45], it is not safe to proceed with naltrexone administration at least until the urine drug screen contains none of these opioid drugs or medications. If the urine drug screen is positive for any of these compounds, postpone initial naltrexone administration for 24–48 h and recheck the urine drug screen at that time and do not proceed with naltrexone until it is negative for opioid drugs or medications.

If the urine drug screen is negative for opioids, oxycodone, methadone, and buprenorphine, and the patient can confirm by history that the patient has not had short acting opioids for a minimum of 3–6 days or methadone or buprenorphine for a minimum of 7–10 days, it may be safe to begin naltrexone administration [45].

If there is any doubt about whether the patient is fully withdrawn from opioids and free of physiologic dependence, it is often prudent to consider doing a naloxone challenge [45, 46]. Naloxone, like naltrexone, is a competitive antagonist at the mu-opioid receptor and will also cause precipitated withdrawal in someone with physiologic dependence [46]. However, naloxone has a short half-life, about 60 min [47]. If precipitated withdrawal results from naloxone administration, it will only last a few hours. Oral naltrexone has a half-life of 4 h, and its active metabolite, 6-beta naltrexol has a half-life of 13 h [48]. Precipitated withdrawal from naltrexone can thus last 24 h or longer. Use the following procedure to do the naloxone challenge:

- Ask if patient has any opioid withdrawal symptoms. If symptoms are present, do not proceed with challenge and re-evaluate in 24–48 h.
- Observe patient for signs of opioid withdrawal. If signs are present, do not proceed with challenge and re-evaluate in 24–48 h.
- If no signs or symptoms are present, obtain baseline vital signs.
- A total dosage of 0.8 mg naloxone must be administered in one of 3 ways:
  1. 0.8 mg IM in deltoid and observe 45 min.
  2. 0.8 mg sub q in any extremity and observe 45 min.
  3. 0.2 mg IV push. Wait 30 s and observe. If no signs or symptoms of opioid withdrawal, administer remaining 0.6 mg IV push and observe 20 min.

If any elevations in pulse rate or blood pressure occur, or if any signs or symptoms of opioid withdrawal emerge, the patient has failed the naloxone challenge. Repeat in 24–48 h assuming no inter-current relapse to opioid use has occurred.

If no elevations in pulse rate or blood pressure occur, and if no signs or symptoms of opioid withdrawal emerge, the patient has passed the naloxone challenge and can proceed to naltrexone ingestion or administration.

Once it has been determined that the naloxone challenge is not necessary, or the patient has passed the challenge, the next step is to determine the level of support the patient has so that a decision can be made about starting oral versus extended release injectable naltrexone (XR-NTX). If the patient has external contingencies such as legal, professional, or family consequences if a relapse to opioid use occurs; or a high level of internal motivation and a mechanism for monitored ingestion of oral medication by program staff, a reliable family member, or a pharmacy, oral naltrexone may be an effective intervention [49, 50]. Monitored oral naltrexone can be started at 50 mg orally once daily or a 3 days per week dosing schedule of 100 mg, 100 mg, 150 mg orally on Mon/Wed/Fri, respectively, with medical management and/or referral to a treatment program and/or mutual help groups. It is reasonable to recheck liver tests within approximately 12 weeks and then as clinically indicated.

If the patient is non-adherent or otherwise fails oral naltrexone and exhibits an intermittent pattern of opioid use with no physiologic dependence and is otherwise tolerating oral naltrexone, a reasonable plan is to switch to XR-NTX. Similarly, if the patient does not have an adequate support system in place, it makes more sense to begin treatment with XR-NTX as described below.

If patient has inadequate supports but refuses XR-NTX and wants oral NTX, then use shared decision-making regarding likelihood of stopping oral NTX and relapsing and consider buprenorphine or methadone as better alternatives to oral NTX. If the patient still wants oral NTX, try to get agreement that if patient tries and fails, patient will consider these other options. Begin oral NTX with very close clinical monitoring and frequent urine drug screens. If patient fails, again present other options.

If the patient is non-adherent or otherwise fails oral naltrexone, exhibits a regular pattern of illicit opioid use, and now has developed physiologic dependence, it makes sense to switch the patient to buprenorphine treatment or refer for methadone treatment.

If the patient and provider agree to start XR-NTX, begin XR-NTX as a 380 mg IM injection in the gluteal muscle [51]. Start medical management including periodic urine drug screens and/or referral to treatment program and/or mutual help groups. Schedule an appointment for the next injection in 4 weeks. Give the next injection in the contralateral gluteal muscle and continue to alternate sites with each subsequent injection. Recheck liver tests in 12 weeks and then as clinically indicated.

If the patient is non-adherent or otherwise fails XR-NTX with an intermittent pattern of opioid use but still no physiologic dependence, a reasonable plan is to continue XR-NTX and intensify behavioral interventions. Alternatively, one can begin buprenorphine treatment or refer to methadone treatment.

If the patient is non-adherent (e.g., does not return for subsequent injections) or otherwise fails XR-NTX with regular pattern of illicit opioid use and now has physiologic dependence, it makes sense to switch the patient to buprenorphine treatment or refer for methadone treatment.

Managing side effects:

- In addition to the very low risk of liver injury from oral naltrexone, common side effects of both formulations of naltrexone include nausea and/or vomiting, dizziness, headache, insomnia, nervousness, and lethargy.
- Side effects frequently wane over days or a few weeks.
- Nausea can often be effectively managed with a short course of an antiemetic.
- Injection site reactions are a common side effect of the extended release injection formulation.
  - Most are benign and resolve with conservative measures such as non-steroidal anti-inflammatory medication and alternating hot and cold packs.
  - Rarely a sterile abscess may require surgical attention or a non-sterile abscess may require antibiotics and/or incision and drainage.

## Systems for referral for treatment and additional services

When referral for treatment and additional services is considered, it is recommended that several items be addressed. First, the type and severity of each substance use disorder should be identified. For each disorder, it is essential to determine if the patient is ready to undergo more formal assessment and possible referral for treatment [52].

If the patient is not ready to undergo more formal assessment and possible referral for treatment, they should be offered further contact. The clinician should present feedback and their concerns about the patient’s health if the patient is interested and desires to discuss. Lastly the clinician may offer information with referral options (e.g., written material, internet resources).

If the patient is unsure if he/she is ready to undergo more formal assessment and possible referral for treatment, the clinician should attempt to facilitate the patient’s ability to name the problem by discussing pros and cons of change (acceptance of treatment). If possible, the clinician should attempt to determine the source of the patient’s ambivalence.

If the patient is ready to undergo more formal assessment and possible referral for treatment, the clinician should confirm the patient’s actual ability/willingness to access a specific resource.

When establishing a system to support clinicians who may refer a patient for treatment and additional services, the practice should identify required resources to address active addiction related clinical problems or situations. Based on the local resources available, the practice should determine specific steps the clinician should follow to ensure appropriate and efficient referral. These steps should be explicitly defined, standardized, streamlined, and documented as standard operating principles for the practice.

Once patient readiness, willingness, and ability have been assessed and confirmed, a placement assessment employing a standardized referral assessment is recommended. The ASAM Criteria [53] is an accepted and validated tool for complete intake and assessment that can facilitate and guide selection of appropriate referral destinations and successful referral to treatment. The core dimensions encompassed by the ASAM Criteria include: (1) Acute Intoxication and/or Withdrawal Potential; (2) Biomedical Conditions and Complications, (3) Emotional, Behavioral, or Cognitive Problems and Complications, (4) Readiness to Change, (5) Relapse, Continued Use, or Continued Problem Potential, and (6) Recovery Environment. Other placement criteria may exist or may be required based on the patient’s insurance coverage.

While ASAM criteria are recognized and broadly accepted, especially in substance use treatment settings, implementing them in primary care settings, even those that are well resourced may be problematic. More pragmatic approaches for assessment and placement, such as clinical judgment of a trained clinician may be necessary and appropriate.

## Workflow (systems) issues

A practice system must determine which personnel (Clinician, Social Worker, etc.) will perform each of the following key tasks based on the local personnel and workflow:

1. Make and/or confirm the diagnosis of a SUD.
2. Make the initial decision to assess the severity of the patient’s SUD.
3. Present the indication for treatment to the patient and introduce the idea of pursuing treatment.
4. Perform an assessment of the patient’s current readiness for treatment.
5. Discuss potential options for treatment with the patient.
6. Clarify the patient’s actual ability and/or willingness for referral to treatment based on appropriate and acceptable options.
7. Make and follow through with the referral for the patient.

When establishing such a system, it is important to determine if the system has the capacity (in the form of a trained individual such as social worker, etc.) available for formal intake and assessment and referral on-site.

If a trained individual is available on-site, then the patient should be referred to that individual to perform formal intake, assessment and referral using a tool (e.g., ASAM Criteria) or clinical judgment to select an appropriate referral destination. If a trained individual is not available, then the patient should be referred to outside resources to perform formal intake, assessment and referral. It should be determined who will actually make this referral and under what circumstances.

## Conclusion

The current epidemic of opioid misuse and overdose has greatly outpaced the capacity of specialized treatment settings to manage it. Integration of OUD screening, assessment, and treatment within primary care systems could potentially help stem the tide of this epidemic, by increasing access to evidence-based care. The NIDA CCTN CDS Working Group describes a semi-structured outline of clinical decision support that may facilitate this process. Site-specific adaptation will be needed to fit the workflow and roles of healthcare personnel at specific sites. The NIDA CTN has funded a pilot study, CTN-0076ot: Clinical Decision Support for Opioid Use Disorders in Medical Settings: Pilot Usability Testing in an EMR (COMPUTE), to program the Working Group’s algorithm into a web-based CDS insert in an electronic health record. This pilot has been completed with usability testing results forthcoming. A NIDA CTN NIH HEAL Initiative-funded multisite clinic randomized trial of the CDS (CTN-0095: COMPUTE 2.0 Clinic-Randomized Trial of Clinical Decision Support for Opioid Use Disorders in Medical Settings) CDS will begin in 2020.

CDS tools can help health systems improve the quality of healthcare delivery. The utility of these tools lies in their ability to streamline and adapt complex treatment algorithms into targeted recommendations for the patient at hand by mining extant and real-time data in the electronic health record. Ultimately, to be effective, a CDS tool must be useful to the provider, patient specific, and adaptive to local needs (e.g., workflows, referral pathways, etc.) and/or changes in treatment standards (e.g., new drug formulations, evolving safety information, etc.).

## Supplementary information

**Additional file 1.** Opioid use disorder clinical algorithm.

## Data Availability

Data sharing is not applicable to this article as no datasets were generated or analyzed during the current study.

## Abbreviations

ASAM: American Society of Addiction Medicine
BUP: buprenorphine
CTN: NIDA Clinical Trials Network
EHR: electronic health record
CDS: clinical decision support tool
CMS: Centers for Medicare and Medicaid Services
CPDD: College on Problems of Drug Dependence
DAST-10: drug abuse screening test
DSM: Diagnostic and Statistical Manual of Mental Disorders published by the American Psychiatric Association
HBV: hepatitis B
HCV: hepatitis C
HARK: instrument to identify intimate partner violence
HITS: domestic violence screening tool
HIV: human immunodeficiency virus
HEDIS: Healthcare Effectiveness Data and Information Set
HRSA: Health Resources & Services Administration
ICD: International Classification of Diseases
IDU: injecting drug use
INEBRIA: International Network on Brief Interventions for Alcohol and Other Drugs
IOM: Institute of Medicine
MOUD: medications for opioid use disorder
ONC: Office of the National Coordinator for Health Information Technology
OBOT-B: Office-Based Opioid Treatment with Buprenorphine
OUD: opioid use disorder
OTP: opioid treatment program
NIDA: National Institute on Drug Abuse
NTX: naltrexone
NX: naloxone
PHQ-2/9: Patient Health Questionnaire -2 or -9
SAMHSA: Substance Abuse and Mental Health Services Administration
SUD: substance use disorder
STaT: intimate partner screening tool
STIs: sexually transmitted infections
TAPS: tobacco, alcohol, prescription medication and other substances use screening tool
TB: tuberculosis
USPSTF: US Preventative Services Task Force
VA/DoD: Department of Veterans Affairs/US Department of Defense
WHO ASSIST: World Health Organization Alcohol, Smoking and Substance Involvement Screening Test
XR-NTX: extended-release naltrexone
