# Supplementary material for: Developing a clinical decision support for opioid use disorders: a NIDA center for the clinical trials network working group report
Source: Addict Sci Clin Pract. 2020 Jan 16;15:4. doi: 10.1186/s13722-020-0180-2 (PMC6966783; doi:10.1186/s13722-020-0180-2)
Supplement: Supplementary file 1 — Additional file 1. Opioid use disorder clinical algorithm. [file 13722_2020_180_MOESM1_ESM.pdf]

Figure 1 in Multiple Parts Denoted as Pages 1 – 9

Clinical Decision Support for Opioid Use Disorders (Short Version)

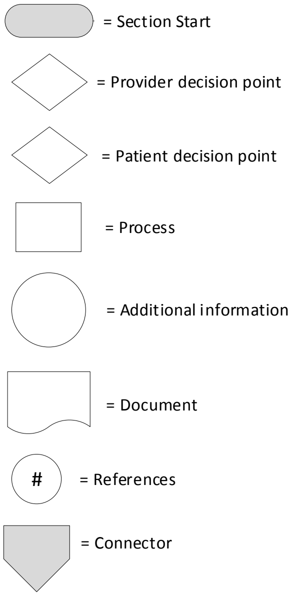

Abbreviations:

DSM = APA Diagnostic and Statistical Manual of Mental Disorders
OUD = Opioid Use Disorder
NX = Naloxone
NTX = Naltrexone
BUP = Buprenorphine
OBOT-B = Office-Based Opioid Treatment with Buprenorphine
MOUD = Medications for Opioid Use Disorder

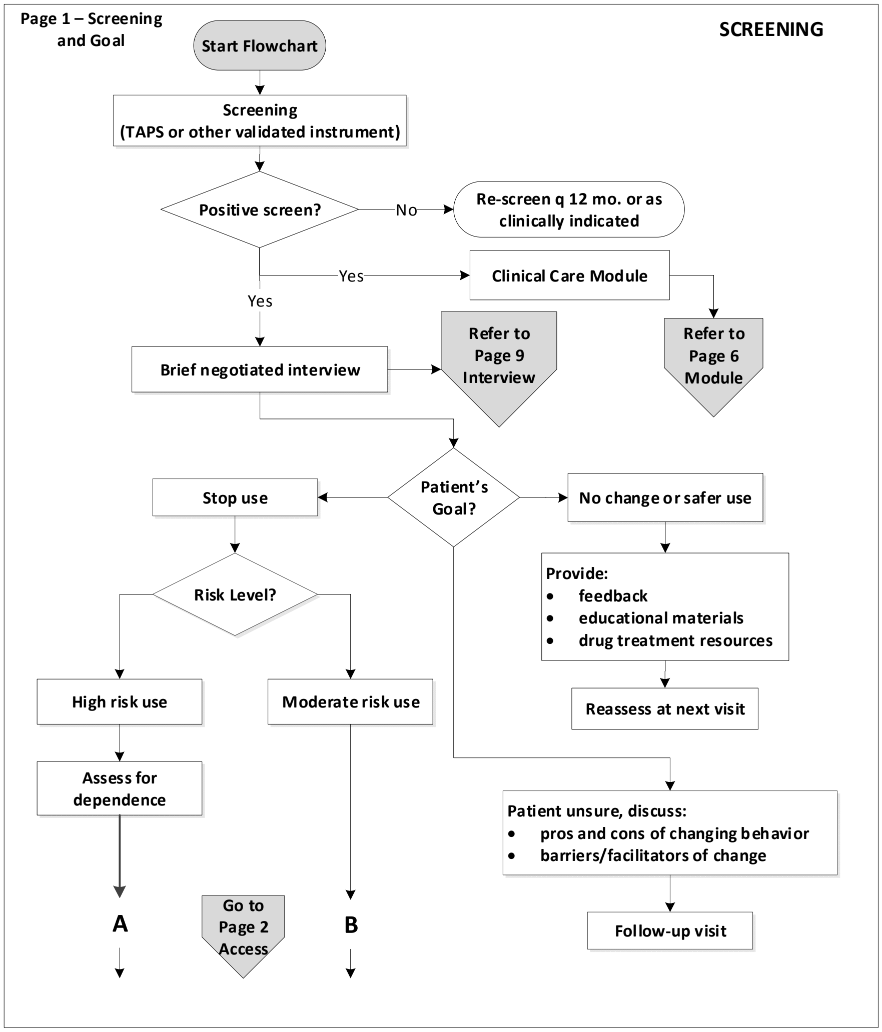

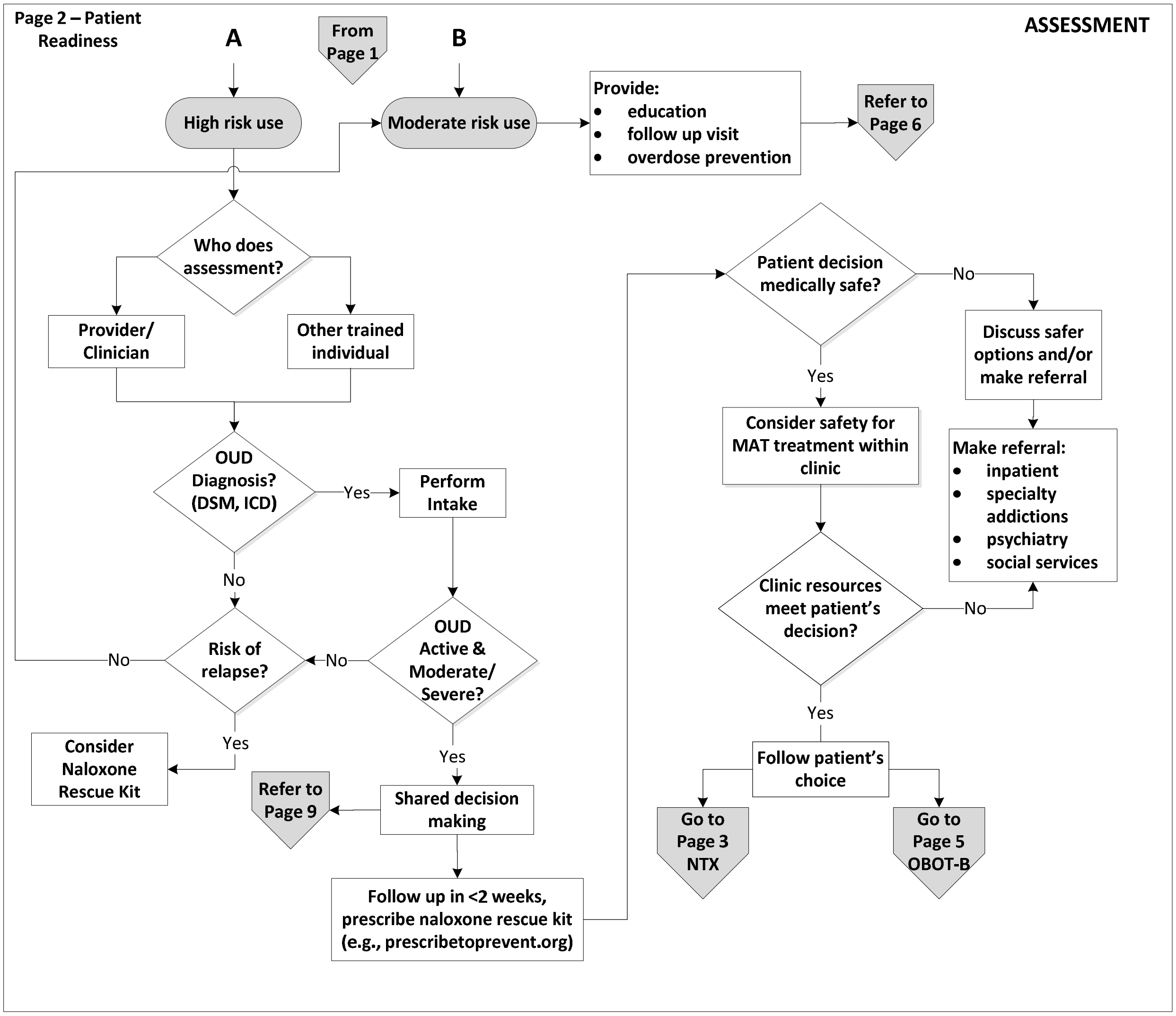
Page 2 – Patient Readiness
ASSESSMENT
A
From Page 1
B
High risk use
Moderate risk use
Provide:
• education
• follow up visit
• overdose prevention
Refer to Page 6
Who does assessment?
Provider/ Clinician
Other trained individual
OUD Diagnosis? (DSM, ICD)
Yes
Perform Intake
No
Risk of relapse?
No
OUD Active & Moderate/ Severe?
No
Yes
Consider Naloxone Rescue Kit
Yes
Refer to Page 9
Shared decision making
Follow up in <2 weeks, prescribe naloxone rescue kit (e.g., prescribetoprevent.org)
Patient decision medically safe?
No
Discuss safer options and/or make referral
Yes
Consider safety for MAT treatment within clinic
Make referral:
• inpatient
• specialty addictions
• psychiatry
• social services
Clinic resources meet patient's decision?
No
Yes
Follow patient's choice
Go to Page 3 NTX
Go to Page 5 OBOT-B

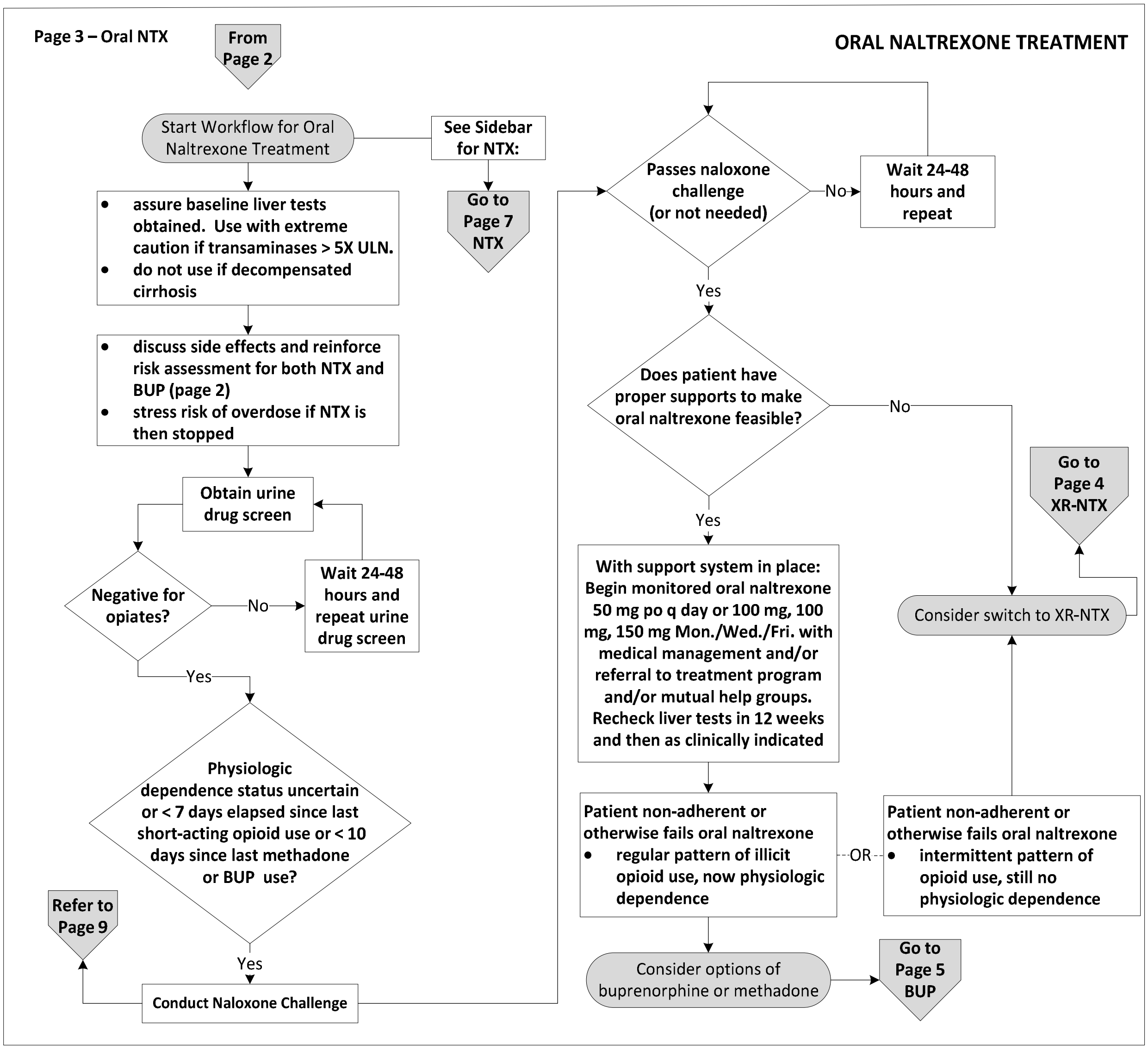

Page 3 – Oral NTX
From Page 2
ORAL NALTREXONE TREATMENT
Start Workflow for Oral Naltrexone Treatment
See Sidebar for NTX:
Go to Page 7 NTX
• assure baseline liver tests obtained. Use with extreme caution if transaminases > 5X ULN.
• do not use if decompensated cirrhosis
• discuss side effects and reinforce risk assessment for both NTX and BUP (page 2)
• stress risk of overdose if NTX is then stopped
Obtain urine drug screen
Negative for opiates?
No
Wait 24-48 hours and repeat urine drug screen
Yes
Physiologic dependence status uncertain or < 7 days elapsed since last short-acting opioid use or < 10 days since last methadone or BUP use?
Yes
Refer to Page 9
Conduct Naloxone Challenge
Passes naloxone challenge (or not needed)
No
Wait 24-48 hours and repeat
Yes
Does patient have proper supports to make oral naltrexone feasible?
No
Yes
With support system in place: Begin monitored oral naltrexone 50 mg po q day or 100 mg, 100 mg, 150 mg Mon./Wed./Fri. with medical management and/or referral to treatment program and/or mutual help groups. Recheck liver tests in 12 weeks and then as clinically indicated
Go to Page 4 XR-NTX
Consider switch to XR-NTX
Patient non-adherent or otherwise fails oral naltrexone
• regular pattern of illicit opioid use, now physiologic dependence
OR
Patient non-adherent or otherwise fails oral naltrexone
• intermittent pattern of opioid use, still no physiologic dependence
Consider options of buprenorphine or methadone
Go to Page 5 BUP

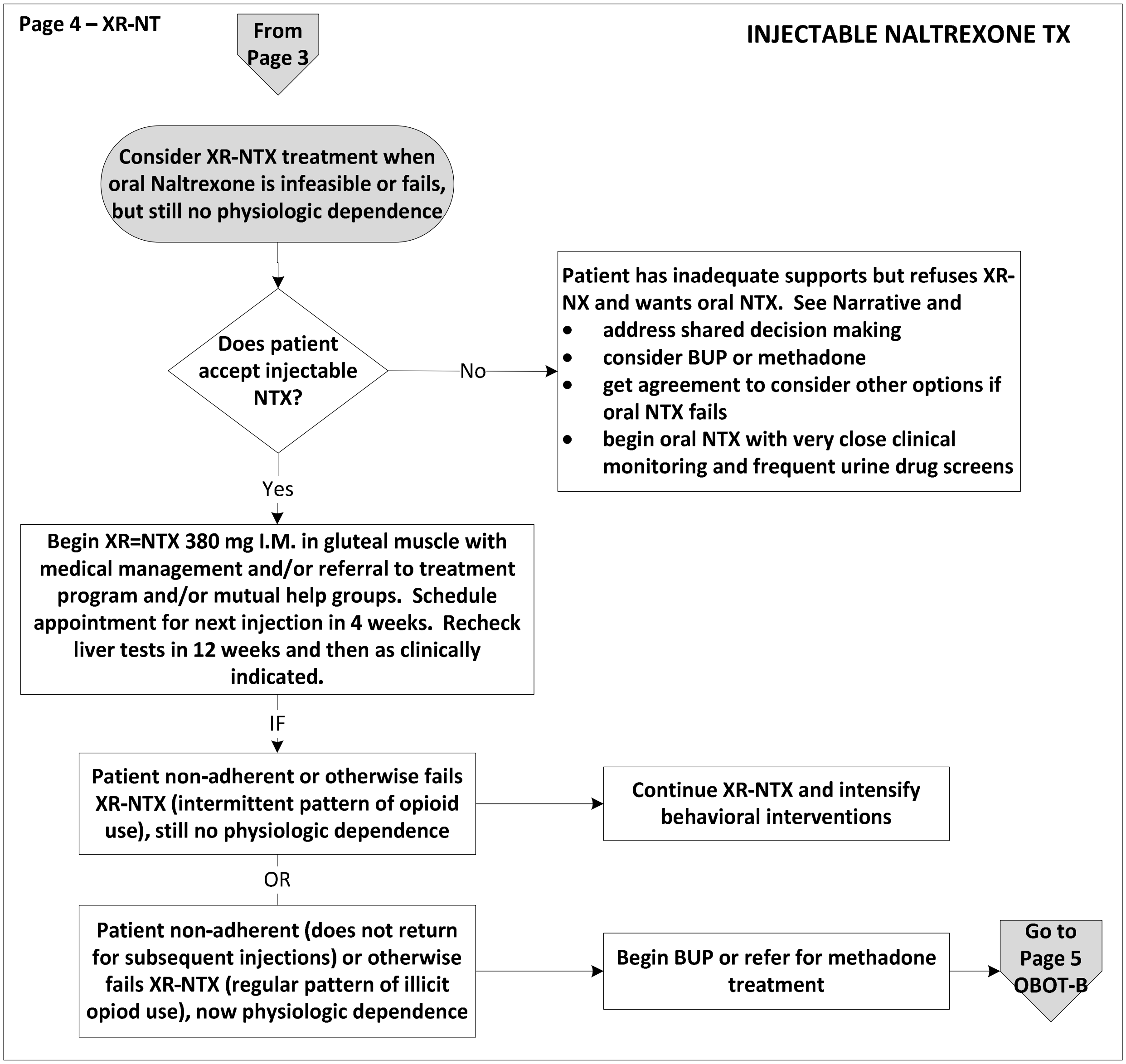
Page 4 – XR-NT
From Page 3
INJECTABLE NALTREXONE TX
Consider XR-NTX treatment when oral Naltrexone is infeasible or fails, but still no physiologic dependence
Does patient accept injectable NTX?
No
Patient has inadequate supports but refuses XR-NX and wants oral NTX. See Narrative and
• address shared decision making
• consider BUP or methadone
• get agreement to consider other options if oral NTX fails
• begin oral NTX with very close clinical monitoring and frequent urine drug screens
Yes
Begin XR=NTX 380 mg I.M. in gluteal muscle with medical management and/or referral to treatment program and/or mutual help groups. Schedule appointment for next injection in 4 weeks. Recheck liver tests in 12 weeks and then as clinically indicated.
IF
Patient non-adherent or otherwise fails XR-NTX (intermittent pattern of opioid use), still no physiologic dependence
Continue XR-NTX and intensify behavioral interventions
OR
Patient non-adherent (does not return for subsequent injections) or otherwise fails XR-NTX (regular pattern of illicit opiod use), now physiologic dependence
Begin BUP or refer for methadone treatment
Go to Page 5 OBOT-B

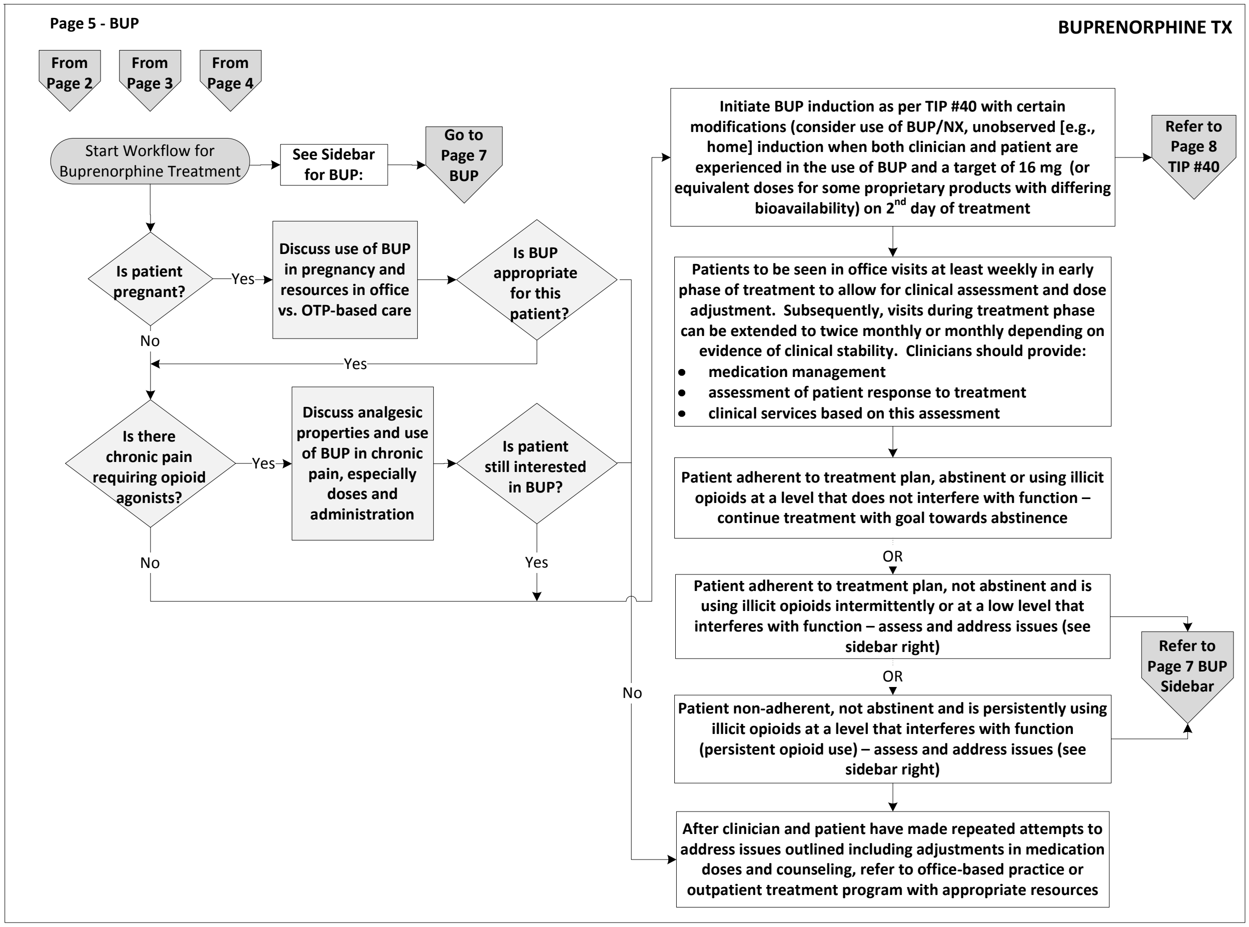
Page 5 - BUP
BUPRENORPHINE TX
From Page 2
From Page 3
From Page 4
Start Workflow for Buprenorphine Treatment
See Sidebar for BUP:
Go to Page 7 BUP
Is patient pregnant?
Yes
No
Discuss use of BUP in pregnancy and resources in office vs. OTP-based care
Is BUP appropriate for this patient?
Yes
Is there chronic pain requiring opioid agonists?
Yes
Discuss analgesic properties and use of BUP in chronic pain, especially doses and administration
Is patient still interested in BUP?
No
Yes
No
Initiate BUP induction as per TIP #40 with certain modifications (consider use of BUP/NX, unobserved [e.g., home] induction when both clinician and patient are experienced in the use of BUP and a target of 16 mg (or equivalent doses for some proprietary products with differing bioavailability) on 2nd day of treatment
Refer to Page 8 TIP #40
Patients to be seen in office visits at least weekly in early phase of treatment to allow for clinical assessment and dose adjustment. Subsequently, visits during treatment phase can be extended to twice monthly or monthly depending on evidence of clinical stability. Clinicians should provide:
• medication management
• assessment of patient response to treatment
• clinical services based on this assessment
Patient adherent to treatment plan, abstinent or using illicit opioids at a level that does not interfere with function – continue treatment with goal towards abstinence
OR
Patient adherent to treatment plan, not abstinent and is using illicit opioids intermittently or at a low level that interferes with function – assess and address issues (see sidebar right)
OR
Patient non-adherent, not abstinent and is persistently using illicit opioids at a level that interferes with function (persistent opioid use) – assess and address issues (see sidebar right)
Refer to Page 7 BUP Sidebar
After clinician and patient have made repeated attempts to address issues outlined including adjustments in medication doses and counseling, refer to office-based practice or outpatient treatment program with appropriate resources

Page 6 - Clinical Care Module

From Page 1

From Page 2

Clinical Care Module - clinical actions that are independent of the treatment decision

- review screening results for tobacco, alcohol, other drug use
- assess lifetime/current IDU, counseling on safer injection
- ask about drug treatment history
- screening for infectious diseases: HIV, HBV, HCV, STIs, TB
- screening for common health and mental health problems: chronic pain, depression, anxiety
- screening for pregnancy
- review of prescribed medications with potentially harmful interactions: sedatives, opioids
- check prescription drug monitoring program
- obtain/Review urine toxicology results
- overdose education, provision of naloxone kit
- vaccination for HAV, HBV, tetanus, influenza, pneumococcal pneumonia (as indicated)

SIDEBARS FOR NTX & BUP

From Page 3

Naltrexone Sidebar:

Assure:
- adequate resources (e.g., insurance coverage for medication, visits, counseling, urine and blood testing)
- ability to safely store medication
- baseline liver tests obtained
- no contraindication to use of NTX
- confidentiality

Consider:
- comorbid substance use/disorders
- benzodiazepine use disorder
- untreated psychiatric comorbidity
- visit frequency
- history of adherence and of diversion
- comorbid chronic pain

Assess and address:
- medication adherence
- triggers
- comorbid substance use
- psychosocial counseling needs

From Page 5
From Page 7

Buprenorphine Sidebar:

Assure:
- adequate resources (e.g., insurance coverage for medication, visits, counseling, urine and blood testing)
- ability to safely store medication
- baseline liver tests obtained
- no contraindication to use of BUP
- confidentiality

Consider:
- comorbid substance use/disorders
- benzodiazepine use disorder
- untreated psychiatric comorbidity
- visit frequency
- history of adherence and of diversion
- comorbid chronic pain

Assess and address:
- medication adherence
- dose to assure cross tolerance and narcotic blockade
- triggers
- comorbid substance use
- psychosocial counseling needs

Page 8 – ASAM Guidelines

The ASAM National Practice Guideline for the Use of Medications in the Treatment of Addiction Involving Opioid Use

http://www.asam.org/docs/default-source/practice-support/guidelines-and-consensus-docs/national-practice-guideline.pdf

ADDITIONAL RESOURCES

From Page 1

Brief Negotiated Interview
- raise the subject
- provide feedback on screening score
- recommend cessation/ reduction
- assess readiness/confidence
- ask patient why their readiness/confidence rating is not lower
- ask patient if they are ready to set a goal for changing their use

From Page 2

Shared decision making
- explain the diagnosis of OUD
- reduced mortality
- reduced HIV and HCV
- reduced hospitalizations
- improved QOL
- distribute information (e.g., SAMHSA facts on BUP, NTX, MOUD)
- probe for goals
  - stop or reduce use
  - work with MD or community organization
  - counseling referral
- review risk/been on meds
  - starting, stopping
  - cost
  - side effects
    - mental health
    - sex
    - sleep
    - bone/teeth
    - weight
    - constipation
  - drug interactions
  - frequency of dosing
  - effects of pain
- pursue treatment plan

Treatment Plan
- medication adherence
- confidentiality
- patient and provider expectations (controlled substance agreement/contract)
- psychosocial counseling (medical management at minimum)
- consideration of referral to mutual help (e.g., A.A., N.A.)
- urine toxicology monitoring
- pregnancy monitoring
- assessment of function
  - social
  - employment/financial
  - substance use
  - psychiatric
  - medical
  - legal

From Page 3

How to conduct Naloxone Challenge
- ask if patient has any opioid withdrawl symptoms. If symptoms are present do not proceed with challenge.
- observe patient for signs of opioid withdrawl. If signs are present do not proceed with challenge.
- if no signs or symptoms are present, obtain baseline vital signs.
- a total dosage of 0.8 mg (2 ampules) naloxone must be administered in one of 3 ways:
- 0.8 mg IM in deltoid observe 45 minutes
- 0.8 mg sub q in ay extremity observe 45 minutes
- 0.2 mg IV push. Wait 30 seconds and observe. If no signs or symptoms of opiod withdrawal, administer remaining 0.6 mg IV push and observe 20 minutes
- if any elevations in pulse rate or blood pressure occur, or if any signs or symptoms of opioid withdrawal emerge, the patient has failed naloxone challenge. Repeat in 24-48 hours.
- if no elevations in pulse rate or blood pressure occur, and if no signs or symptoms of opioid withdrawal emerge, the patient has passed the naloxone challenge and can proceed to naltrexone administration.
